# Lung abnormalities on computed tomography of Vietnamese patients with COVID-19 and the association with medical variables

**DOI:** 10.1016/j.ijregi.2024.01.006

**Published:** 2024-01-19

**Authors:** Cong Dien Trinh, Van Nam Le, Van Nguyen Bang Le, Ngoc Thach Pham, Van Duyet Le

**Affiliations:** 1 Departments of Infectious Disease, Military Hospital 103, Hanoi, Vietnam; 2 Luong The Vinh High School, Hanoi, Vietnam; 3 Micobiology and Molecular Biology Department, National Hospital for Tropical Diseases, Hanoi, Vietnam

**Keywords:** COVID-19, Lung injury, Lung CT abnormality, CT scan, Lung involvement

## Abstract

**Objectives:**

Patients with COVID-19 may experience a lung injury without presenting clinical symptoms. Early detection of lung injury in patients with COVID-19 is required to enhance prediction and prevent severe progression.

**Methods:**

Lung lesions in patients with COVID-19 were defined using the Fleischner Society terminology. Chest computed tomography lesions and their correlation with demographic characteristics and medical variables were identified.

**Results:**

Patients with mild and moderate COVID-19 had up to 45% lung injuries, whereas critical patients had 55%. However, patients with mild and moderate COVID-19 typically had low-level lung injuries. Ground-glass (68.1%), consolidation (48.8%), opacity (36.3%), and nodular (6.9%) lung lesions were the most prevalent in patients with COVID-19. Patients with COVID-19 infected with the Delta variant had worse lung injury than those infected with the Alpha and Omicron. People vaccinated with ≥2 doses showed a lower risk of lung injury than those vaccinated with <1 dose. Patients <18 years old were less likely to have a lung injury than patients >18 years old. The treatment outcomes were unaffected by the severity of the lung injury.

**Conclusion:**

Patients with mild COVID-19 had a similar risk of lung injury as patients with severe COVID-19. Thus, using chest computed tomography to detect lung injury can enhance the treatment outcomes and reduce the patient's risk of pulmonary complications.

## Highlights

• Lung injury was present in 57.6% of patients with COVID-19.
• The most prevalent were ground-glass, consolidation, opacity, and nodular lung lesions.
• Patients aged ≥60 years with co-morbidities and severity had a higher rate of lung injury.
• Lung injury ≥25% was related with older age, the Delta variant, and severity.
• A total of 100% of patients with lung injury died.

## Introduction

SARS-CoV-2 is a coronavirus that causes severe acute respiratory syndrome. It was detected for the first time in December 2019 in Wuhan, China and was formally designated by the International Committee for the Classification of Viruses in February 2020 [1]. As of February 3, 2023, SARS-CoV-2 was responsible for the global COVID-19 pandemic, which resulted in over 754 million infections and over 6 million deaths [2]. Because there have been four outbreaks in Vietnam since the first case was reported on January 23, 2020, the spread of SARS-CoV-2 with the emergence of new variants is currently underway and has had a significant negative impact on many aspects of social life [3,4].

Patients infected with SARS-CoV-2 can present with a wide range of clinical symptoms, ranging from no symptoms (mild) to moderate viral pneumonia to severe respiratory failure, multi-organ system dysfunction, sepsis, and death. Mild COVID-19 is diagnosed in individuals with or without non-specific clinical symptoms, such as fever, dry cough, sore throat, nasal congestion, fatigue, muscle aches, and no signs of pneumonia. A moderate level is characterized as pneumonia (fever, cough, difficulty breathing, and rapid breathing) with no evidence of severe pneumonia and oxygen saturation (SpO_2_) >90% when inhaling air. Symptoms of severe disease include fever, SpO_2_ <90%, respiratory failure, consciousness issues, and convulsions [5]. When tested for the virus, up to 40-45% of those infected are asymptomatic, and many of them continue to be asymptomatic after clearing the virus from their upper respiratory tract and being able to infect others [6]. Other patients developed symptoms between 2 and 14 days after exposure, with a 5-day average incubation time [7]. Clinical symptoms vary by region, ethnicity, and country worldwide, notably, in the elderly and people with chronic conditions, immunodeficiency, or cancer [8].

Although real-time reverse transcription-polymerase chain reaction (RT-PCR) tests were used to confirm SARS-CoV-2 infection, imaging techniques such as computed tomography (CT) of the chest aid in the diagnosis, severity assessment, identifying complications, and reviewing treatment response [9]. Although CT of the chest is not a definite criterion for diagnosing COVID-19 infection, the lesions found aid in making a diagnosis in the context of clinical presentation and epidemiology. COVID-19 infection is identified by ground glass, pulmonary parenchymal consolidation, stone pavement, and vasodilation, as well as a reversed halo sign in the periphery, bilateral lung, and early lower lesions [10]. Patients with COVID-19 frequently develop acute respiratory distress syndrome, pulmonary embolism due to thrombosis, superinfection pneumonia, and heart failure [11].

CT imaging can accurately differentiate SARS-CoV-2 pneumonia lesions from other viruses [12,13]. The initial CT findings of COVID-19 include bilateral, peripheral, or posterior multilobular ground-glass opacities (GGOs), which are most prevalent in the lower lobes and less common in the middle lobes. Consolidation superimposed over GGOs occurs in a rare number of instances, primarily among the elderly. In the latter stages of the illness, GGOs transform into multifocal consolidation, interlobular and intralobular tract thickening, crazy squamosalization, traction bronchiectasis, pleural thickening, and subpleural bands. Pleural/pericardial effusion, enlarged mediastinal lymph nodes, dental caries, halo sign on CT, and pneumothorax are rare but may occur as pneumonia advances [14]. The majority of patients with COVID-19 did not develop cavernous, destructive lesions. As a result, chest CT is a frequent imaging method used to evaluate lung lesions; the procedure is often simple to conduct and yields speedy results, which aid in COVID-19 diagnosis. Patients are normally assessed by non-contrast CT of the chest, with the exception of pulmonary angiography to detect thrombosis.

In Vietnam, there have been very few reports of lung injury in hospitalized patients with COVID-19, specifically, lung injury caused by the SARS-Cov-2 variant in association with blood oxygen levels and changes in biochemical, hematologic, and therapeutic effects. Therefore, this study aimed to assess the characteristics and rate of lung damage in patients with COVID-19 at three clinical levels—mild, moderate, and severe–using chest CT images to estimate the incidence of lung injury in patients with COVID-19, particularly, those with no or mild symptoms. Furthermore, the study investigated whether factors such as the age of patients; vaccination; and Alpha, Delta, and Omicron variant infection affect the rate and severity of lung injury in patients with COVID-19.

## Methods

### Study population

A total of 278 patients with COVID-19 were treated in hospitals in northern Vietnam before being transferred to National Hospital for Tropical Diseases (NHTD). Clinical evaluation was performed, followed by real-time RT-PCR confirmation, SARS-CoV-2 whole genome sequencing to identify variants, and chest CT to determine lung injury. All clinical and laboratory information was obtained from the patient's medical records.

### Criteria for patient selection and exclusion

- Criteria for selection of patient: 550 patients with COVID-19 were diagnosed with COVID-19 in accordance with the Ministry of Health of Vietnam's guidance (Decision 250/QD-BYT 2022) and treated at the NHTD, of whom 278 patients had a chest CT scan during the study period.
- Patient exclusion criteria: 272 patients were diagnosed with COVID-19, of whom 175 did not receive a CT scan, and 97 patients revealed images of lung injury not caused by COVID-19 (lung tumors, tuberculosis, *etc*.) were excluded.
- CT scan time: When the patient is admitted to the hospital or clinical findings worsen, a CT scan is performed. Patients who underwent many CT scans will receive the most common results from each scan. CT scanning was done within 1-3 days of the onset of symptoms.

### Chest CT scan

Only patients suspected of having pulmonary artery thrombosis will have a CT scan with contrast injection; all others will get a CT scan of the lungs without contrast. Low-dose CT scans, on the other hand, were advised because the patient may require multiple CT scans, minimizing the amount of radiation that is harmful to the patient. Standard-dose CT typically uses the values 130 kVp and 45-162 mA. The radiation exposure dose of a single scan with these parameters is roughly 1.4 mA, which is only one-fifth of the dose of a standard CT scan. The capture was accomplished with a single inhalation. Expiratory imaging, although effective in finding gas traps, increases the radiation dosage but has not been shown to improve COVID-19 monitoring and prognosis.

The patient was positioned supine and a spiral scan of the entire thorax, from the clavicle to the lung base, was performed. During the shot, the following parameters were used: voltage 130 kVp, current 45-162 mA, slice thickness 5 mm with a pitch of 2, average scanning field length 351 ± 37 cm, scan speed 1 second/revolution. The total dose length product achieved a low dosage of 69.81 ± 14.75 mGy × cm on average.

### Assessment of lung injury using CT images

The lesion morphology was described using international standard nomenclature from the Fleischner Society, and the literature on viral pneumonia was evaluated, including ground glass and consolidation. These lesions on a chest CT were assessed, and they included the following:

(i) left lung position (upper and lower lobes) and right lung position (upper, middle, and lower lobes); (ii) distribution (central predominance/peripheral predominance/random and anterior/posterior/diffuse); (iii) extent of lesion (severity scale on CT); (iv) density (consolidation appears as a homogeneous increase in pulmonary parenchymal attenuation that obscures the margins of vessels and airway walls. An air bronchogram may be present; ground glass appears as an area of hazy increased lung opacity, usually extensive, with preservation of bronchial and vascular margins. Nodular is characterized by the presence of innumerable small, rounded opacities that are discrete and range in diameter from 2 to 10 mm. Opacity is known as progressive massive fibrosis and manifests as mass-like lesions, usually bilateral and in the upper lobes); and (v) other signs (crazy paving pattern), interlobular septal thickening, tracheobronchogram, vascular enlargement, bronchiectasis, reversed halo sign, and subpleural fibrous band). In addition, evaluation of extrapulmonary lesions such as large mediastinal lymph nodes (horizontal axis diameter >10 mm), and pleural fluid (yes/no) was also performed.

Severity assessment were performed using CT scans, as proposed by Pan *et al.* [15]. A semi-quantitative CT score was calculated based on the extent of lobar involvement (0: 0%, 1: <5%, 2: 5-25%, 3: 26-50%, 4: 51-75%, 5: >75%, range 0-5, global score 0-25). The resulting global CT score is the sum of each individual lobar score and 0-25.

The score is the amount of parenchymal damage in each lung lobe on both sides:

- zero point if there is no damage,
- one point if the lesion covers 5% of the lung lobe,
- two points if the lesion occupies 5-25% of the lung lobe,
- three points if the lesion covers 26-49% of the lung lobes,
- four points if the lesion occupies 50-75% of the lung lobe, and
- and five points if the lesion occupies >75% of the lung lobe.

CT Severity was assessed on a scale of 0 to 25, with the total score calculated for the five lung lobes.

Three experienced radiologists read and reviewed individual chest CT (Picture Archive and Communication Systems) images. In cases of disagreement, a fourth expert with more than 20 years of expertise will make the final decision.

### Clinical and laboratory collection

We collected all patient data during therapy, including clinical, laboratory, complications, and treatment outcomes. Patient data included age, gender, occupation, residence, and vaccination status; clinical data included respiratory system status, co-morbidities, blood pressure, respiratory rate, SpO_2_, lung injury, and clinical grade. The laboratory data obtained, including the SARS-CoV-2 genome sequence, complications, treatment therapy, treatment duration, and treatment outcomes, were systematically documented.

### Statistical analysis

The collected data were processed and analyzed using SPSS 26.0 software (IBM, New York, NY). Quantitative data are provided as mean ± SD. Unless otherwise specified, qualitative data were provided as a percentage (%) of the total. The χ^2^ test, Fisher's test, and Kruskal–Wallis test were used in the study to assess the lesions and their distribution on chest CT by age group. A *P*-value <0.05 was considered statistically significant. Patients were separated into three age groups: 18 (group 1), 18 to 59 (group 2), and 60 years old (group 3).

## Results

### Demographic and lung CT findings in patients with COVID-19

**Figure 1:**
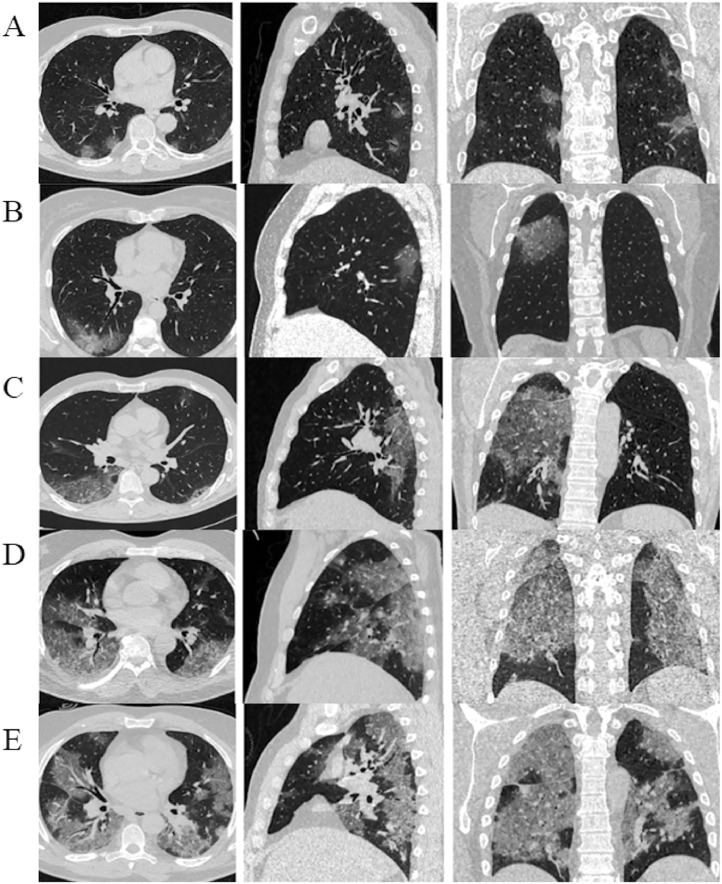
Chest computed tomography images of patients with COVID-19 with varying degrees of lung involvement. A: <10% of involvement (one point), B: 10-25% of involvement (two points), C: 26-49% of involvement (three points), D: 50-75% of involvement (four points), and E: >75% of involvement (five points), respectively.

On chest CT, 160 patients with COVID-19 (57.6%) had lung damage, whereas the rate of severe lung damage (>50% involvement) was only 6.3% (Table 1, Figure 1). Lung injury features include ground glass, nodular, consolidation, and opacity, with ground glass having 68.1% and nodular having 6.9% (Table 1). The lobe distribution has a 72.5-91.9% incidence of lung injuries, whereas the anterior and posterior distributions have a 5.0-64.4% incidence. Only 10.6% of 160 patients with COVID-19 with lung abnormalities on CT scans experienced pleural effusion complications (Table 1).

**Table 1:** Demography, computed tomography findings, and complications of 278 patients with COVID-19.

Characteristics		n (%)
***Gender (n = 278)***		
Male		149 (53.6)
Female		129 (46.4)
***Chest computed tomography of lung injury (n = 278)***		
Lung injury		160 (57.6)
None injury		118 (42.4)
***Lung injury features (n = 160)***		
Ground glass		109 (68.1)
Nodular		11 (6.9)
Consolidation		78 (48.8)
Opacity		58 (36.3)
***Level of lung injury (n = 160)***		
< 10%		67 (41.9)
10 - 25%		73 (45.6)
25 - 50%		10 (6.3)
> 50%		10 (6.3)
***Location of lung injury (n = 160)***		
Side distribution	Right	14 (8.8)
	Left	15 (9.4)
	Both sides	131 (81.8)
Anterior, posterior distribution	Anterior	8 (5.0)
	Posterior	49 (30.6)
	Diffuse	103 (64.4)
Horizontal distribution	Central	25 (15.6)
	Peripheral	43 (26.9)
	Central + peripheral	92 (57.5)
Lobe distribution	Right upper lobe	116 (72.5)
	Right middle lobe	136 (85.0)
	Right lower lobe	130 (81.3)
	Left upper lobe	124 (77.5)
	Left lower lobe	147 (91.9)
***Complication (n = 160)***		
None		143 (89.4)
Pneumothorax		0 (0.0)
Pleural effusion		17 (10.6)

### Pulmonary involvement of all abnormalities

On CT scans, there is no statistically significant difference in the level of lung injury between the elderly (≥60 years old) and the young (Table 2). When the level of lung injury with SARS-CoV-2 variants was compared, it was found that the Delta-infected group had 51% of patients with damage ranging from 10% to 25%, whereas the Alpha-infected group had 15.4% and 11.5% incidence rates, with damage 25-50% and >50%, respectively, with a statistically significant difference. There was no difference in the level of injury with pulmonary involvement of all abnormalities >50%. When the amount of injury was compared with the vaccination status, it was found that the group vaccinated with a complete dosage (two doses) had 5.3% of patients with injuries >50%, whereas the group that did not receive a full dose had 17.5%; however, the difference was not evident. The number of patients with lung injuries of 10-25% was 63.2% in the full-dose vaccination group, whereas the groups that received either one dose or none at all had 47.1% and 39.0%, respectively. Patients with lung damage <10% who did not require oxygen support experienced 61.8%, whereas those who did had 27.9% and 25%, respectively. In addition, the proportion of patients requiring oxygen assistance (particularly, artificial breathing) was 11.8% in the group with a more severe lung injury (>50% pulmonary involvement of all abnormalities). The extent of lung damage also corresponds to the severity of the disease; in patients with mild severity, the rate of serious lung damage is <10%. In addition, the severity of the patients with 10-25% lung injury was 58%, whereas non-severe patients were 30.6% (odds ratio [OR] 3.13 [1.63-6.04]). When the mortality rate was compared with the level of lung injury, it was found that the survival group had 45% of patients with mild lung injury <10% than the 20% in the death group (OR 2.25 [0.92-5.51]), whereas the proportion of patients with severe lung injury (>10%) was 65% in the death group; however, the difference was not statistically significant (this may require larger studies) (Table 2).

**Table 2:** Comparison of demographic and medical variables with the pulmonary involvement of all abnormalities based on lung computed tomography.

		Pulmonary involvement of all abnormalities							
		<10%		10-25%		25-50%		>50%	
		n,%	*P*-value OR (95% CI)	n,%	*P*-value OR (95% CI)	n,%	*P*-value OR (95% CI)	n,%	*P*-value OR (95% CI)
**Age groups** (n, %)	<60 (n = 79)	35 (44.3)	>0.05 1.12 (0.78-1.62)	32 (40.5)	>0.05 0.80 (0.57-1.13)	04 (5.1)	>0.05 1.03 (0.95-1.11)	08 (10.1)	>0.05 0.92 (0.85-1.00)
	≥60 (n = 81)	32 (39.5)		41 (50.6)		06 (7.4)		02 (2.5)	
**Variants** (n, %)	Alpha (n = 26)	13 (50.0)	>0.05	06 (23.1)	**<0.05**	04 (15.4)	**<0.05**	03 (11.5)	<0.05
	Delta (n = 98)	36 (36.7)		50 (51.0)		06 (6.1)		06 (6.1)	
	Omicron (n = 36)	18 (50.0)		17 (47.2)		0 (0.0)		01 (2.8)	
**Vaccine**	None (n = 105)	50 (47.6)	>0.05	41 (39.0)	**<0.05**	09 (8.6)	>0.05	05 (4.0)	>0.05
	1 dose (n = 17)	05 (29.4)		08 (47.1)		01 (5.9)		03 (17.5)	
	≥2 doses (n = 38)	12 (31.6)		24 (63.2)		00 (0.0)		02 (5.3)	
***Oxygen therapies***	Not used (n = 68)	42 (61.8)	**<0.05**	21 (30.9)	**<0.05**	03 (4.4)	>0.05	02 (2.9)	**<0.05**
	Mask cannula (n = 68)	19 (27.9)		37 (54.4)		04 (5.9)		08 (11.8)	
	Ventilator (n = 24)	06 (25.0)		15 (62.5)		03 (12.5)		0 (0.0)	
***Severities***	Non severe (n = 72)	45 (62.5)	**<0.001** **2.00** (1.45-2.76)	22 (30.6)	**<0.001** **3.13** (1.63-6.04)	03 (4.2)	>0.05 0.96 (0.89-1.04)	02 (2.8)	>0.05 0.94 (0.87-1.01)
	Severe (n = 88)	22 (25.0)		51 (58.0)		07 (8.0)		08 (9.1)	
***Treatment outcomes***	Survive (n = 140)	63 (45.0)	**<0.05** **2.25** (0.92-5.51)	60 (42.9)	>0.05 1.63 (0.88-3.02)	08 (5.7)	>0.05 1.63 (0.88-3.02)	09 (6.4)	>0.05 0.99 (0.88-1.10)
	Death (n = 20)	04 (20.0)		13 (65.0)		02 (10.0)		01 (5.0)	

CI, confidence interval; OR, odds ratio.

### Lung CT abnormalities

There were no changes in lung injury features between age groups (≥60 and <60 years old) or between vaccinated and unvaccinated groups. The Alpha-infected group had 92.3% and 30.8% of patients with ground-glass lesions and opacities; the difference was statistically significant with *P-*value <0.05 (Table 3). In addition, consolidation lesions were 64.3% in the Delta group, followed by the Omicron group (30.6%) and the Alpha group (15.4%); the difference was statistically significant. The proportions of opacity lesions in the Delta- and Omicron-infected groups were 42.9% and 41.7%, respectively; and in the Alpha-infected group, it was 3.8%. When lesion characteristics were compared with oxygen support measures, it was found that the proportion of patients with ground-glass lesions and opacities was not different, whereas the group requiring oxygen support and artificial ventilation had 69.1% and 47.1% of patients with consolidation lesions and opacifying bands, and the group that did not require oxygen support had 26.5%; the difference was statistically significant. There was no change in the proportion of patients with ground glass, nodular, or opacities between the severe and mild groups. However, there was a noticeable difference in consolidation lesions, with the severe group accounting for 69.3% of patients with consolidation lesions, whereas the moderate group was 23.6% (OR 2.94 [1.89-4.55], 95% confidence interval). Finally, no variations in the patient's damage features on chest CT scans were detected when comparing the death and survivor groups (Table 3).

**Table 3:** Comparison of demographic and medical variables with lung computed tomography abnormalities.

		Lung computed tomography abnormalities							
		Ground glass		Nodular		Consolidation		Opacity	
		n,%	*P*-value OR (95% CI)	n,%	*P*-value OR (95% CI)	n,%	*P*-value OR (95% CI)	n,%	*P*-value OR (95% CI)
**Age** (n, %)	< 60 (n = 79)	59 (74.7)	> 0.05 1.21 (0.98-1.50)	08 (10.1)	> 0.05 0.99 (0.86-1.02)	37 (46.8)	> 0.05 1.08 (0.80-1.46)	24 (30.4)	> 0.05 1.20 (0.95-1.52)
	≥ 60 (n = 81)	50 (51.7)		03 (3.7)		41 (50.6)		34 (42.0)	
**Variants** (n, %)	Alpha (n=26)	24 (92.3)	**< 0.05**	08 (30.8)	**< 0.001**	04 (15.4)	**< 0.001**	01 (3.8)	**< 0.001**
	Delta (n=98)	66 (67.3)		0 (0.0)		63 (64.3)		42 (42.9)	
	Omicron (n = 36)	19 (52.8)		03 (8.3)		11 (30.6)		15 (41.7)	
**Vaccine**	None (n=105)	72 (68.6)	> 0.05	09 (8.6)	> 0.05	52 (49.5)	> 0.05	34 (32.4)	> 0.05
	1 dose (n = 17)	12 (70.6)		00 (0.0)		11 (64.7)		06 (35.3)	
	≥2 doses (n = 38)	25 (65.8)		02 (5.3)		15 (39.5)		18 (47.4)	
***Oxygen therapies***	Not used (n = 68)	52 (76.5)	> 0.05	08 (11.8)	> 0.05	18 (26.5)	**< 0.001**	18 (26.5)	**< 0.05**
	Mask cannula (n = 68)	40 (58.8)		02 (2.9)		47 (69.1)		32 (47.1)	
	Ventilator (n = 24)	17 (70.8)		01 (4.2)		13 (54.2)		08 (33.3)	
***Severities***	Non severe (n = 72)	54 (75.0)	> 0.05 0.83 (0.68-1.03)	08 (11.1)	> 0.05 1.09 (0.99-1.19)	17 (23.8)	**< 0.001** **2.94** (1.89-4.56)	21 (29.2)	> 0.05 0.82 (0.65-1.03)
	Severe (n = 88)	55 (62.5)		03 (3.4)		61 (69.3)		37 (42.0)	
***Treatment outcomes***	Survive (n = 140)	95 (67.9)	> 0.05 1.07 (0.53-2.18)	10 (7.1)	> 0.05 0.98 (0.88-1.09)	66 (47.1)	> 0.05 1.32 (0.76-2.31)	49 (35.0)	> 0.05 1.18 (0.78-1.79)
	Death (n = 20)	14 (70.0)		01 (5.0)		12 (60.0)		09 (45.0)	

CI, confidence interval; OR, odds ratio.

### Demography and medical variables differed across groups with or without lung injury

Patients >60 years old had 51.9% of lung injury, and those <60 years old had 48.1%, whereas those without lung injury had 25.4% and 74.6%, respectively; the difference was statistically significant. Furthermore, patients with co-morbidities such as lung disease, hypertension, and diabetes have a 6.3%, 35.6%, 16.9%, and 51.9% probability of lung injury, whereas patients who received >2 doses of the vaccine revealed a 23.8% probability of lung injury (Table 4). The proportion of patients requiring oxygen treatment was, likewise, 57.5% in patients with lung injuries, which is similar to the severe and critical patients, showing a higher rate of lung injury than patients with mild and moderate lung injuries. The proportion of death patients with lung injury was 12.5% of the cases, whereas those without lung injury were 0% of the cases; the difference was statistically significant (Table 4).

**Table 4:** Frequencies and percentages of age, and medical variables among the patients with COVID-19 based on lung computed tomography abnormalities.

Characteristics		Lung injury (n = 160)	None injury (n = 118)	Odds ratio (95% confidence interval)	*P*-value (95% confidence interval)
**Age (years old)**	< 18	09 (5.6)	13 (11.0)	**< 0.001**	
	18-59	68 (42.5)	75 (63.6)		
	≥ 60	83 (51.9)	30 (25.4)		
***Co-morbidities***	Lung disease	10 (6.3)	2 (1.7)	**2.62** (0.73-9.34)	**< 0.05**
	Hypertension	57 (35.6)	17 (14.4)	**3.29** (1.79-6.03)	**< 0.001**
	Diabete	27 (16.9)	11 (9.3)	**1.98** (0.94-4.16)	**< 0.05**
	Others	83 (51.9)	34 (28.8)	**2.66** (1.61-4.41)	**< 0.001**
***SARS-CoV-2 variants***	Alpha	26 (16.3)	14 (11.9)	**< 0.05**	
	Delta	98 (61.3)	61 (51.7)		
	Omicron	36 (22.5)	43 (36.4)		
***Vaccination***	None vaccination	105 (65.6)	56 (47.5)	**< 0.001**	
	1 dose	17 (10.6)	07 (5.9)		
	≥ 2 doses	38 (23.8)	55 (46.6)		
***Oxygen therapy***	Not used	68 (42.5)	105 (89.0)	**< 0.001**	
	Mask cannula	68 (42.5)	13 (11.0)		
	Ventilator	24 (15.0)	0 (0.0)		
***Severity***	Mild	06 (3.8)	81 (68.6)	**< 0.001**	
	Moderate	66 (41.2)	28 (23.7)		
	Severe + critical	88 (55.0)	09 (7.6)		
***Treatment outcome***	Survive	140 (87.5)	118 (100.0)	**< 0.001**	
	Death	20 (12.5)	00 (0.0)		

## Discussion

Our findings reveal that patients with mild and moderate COVID-19 have roughly the same risk of lung injury as severe and critical patients. However, the severity of lung injury (as measured by the pulmonary involvement of all abnormalities >10%) in mild and moderate patients is significantly lower than in severe and critical patients. This evidence is consistent with the findings of Mo *et al.* [16], Liu *et al.* [17], and Martínez Chamorro *et al.* [13]. This demonstrates that SARS-CoV-2 causes lung tissue cell destruction even when the patient exhibits no clinical symptoms. In some patients, the advancement of broad lung tissue damage can result in clinical symptoms, such as coughing; trouble breathing; chest pain; shortness of breath; limited mobility; and, possibly, respiratory failure, hypoxia, and multiple organ failure. The findings also demonstrate that mild and moderate patients, as well as persons who have been fully vaccinated, were less likely to develop severe lung damage (pulmonary involvement of all abnormalities index >10%). This is entirely compatible with the patient's clinical symptoms; nevertheless, patients with evidence of lung damage must be continuously monitored and have proper treatment regimens in place to prevent the advancement of lung damage, which is faster and more severe.

The severity score on a CT scan represents the extent of lung injury and has been found to correspond with clinical severity in various investigations. According to Bai *et al.* [18] and Francone *et al.* [19], the severity score on the CT scan of the severe/critical patient group was higher than that of the normal patient group, indicating an increased risk of mortality. Furthermore, Liu *et al.* [20] demonstrated that elderly people are more likely than young people to have multilobar and severe lesions. In our study, older patients had a higher average severity score, which is consistent with previous studies, demonstrating that the elderly had a higher rate of complications and death from COVID-19 than younger patients. Furthermore, the elderly frequently have co-morbidities associated with elevated rates of lung injury in patients with COVID-19. The aging of the body diminishes the resistance of the old, the immune system weakens, and the respiratory system deteriorates over time; thus, when infected with a virus, the sickness progresses faster, becomes more severe, and can cause more problems and mortality. Our findings also show that co-morbidities such as diabetes, hypertension, obesity, cardiovascular disease, neurological illness, cancer, and other disorders are closely connected to the severity of the patients’ diseases, which is consistent with previous studies [21], [22], [23].

The COVID-19 disease produces varied levels of lung damage in the beginning. As it worsens, it can induce acute respiratory distress syndrome. Other organs, including the brain, heart, digestive organs, kidneys, disseminated hypercoagulation, *etc*., are also impacted, either directly or indirectly. A chest CT scan is a common, widely used, non-invasive imaging tool with rapid findings and high value in diagnosing lung damage in the early stages of the disease (some minor lesions are not apparent on X-ray film). CT scan images can be used to assess lung parenchymal damage, respiratory problems, therapy response, and prognosis. Studies conducted by Jalaber *et al.* [24] in France and Bai *et al.* [18] in China and the United States both found differences in chest CT scan features between the groups of patients with COVID-19 pneumonia and those with other viruses.

The findings revealed that the frequencies of ground glass, consolidations, opacities, and nodules varied depending on the stage of COVID-19 disease as well as the clinical symptoms. Furthermore, the patient's lung lesions were mostly detected on both sides of the lungs, which is a common CT imaging technique for patients with COVID-19, as reported in many previous studies. This result differs from the findings of Yasin *et al.* [25], Rousan *et al.* [26], and Rousan *et al.* [26]. However, lung parenchymal consolidation and ground glass were reported as the most common in all of these studies, with variances between studies owing to various patients and SARS-CoV-2 variants. Yasin *et al.* [25] also indicated that the bulk of lung lesions were spread on both sides of the lungs, and there was no bilateral lung-dominant lesion caused by COVID-19.

The rate of ground glass did not change across the three age groups; however, lung parenchymal consolidation is more common in young people, whereas nodularity and opacities are less common in the elderly. Similarly, Chen *et al.* [27] and Sultan *et al.* [28] discovered that elderly people had higher frequencies of lung lesions and ground glass than younger adults. Furthermore, the SARS-CoV-2 variant has a major impact on lung injury in patients with COVID-19, with ground glass occurring often when infected with the Alpha and Delta variants. However, nodules are most common in patients with the Alpha variant, whereas lung parenchymal consolidation and opacities are more common in patients with the Delta and Omicron variants. Ground glass is thought to be the distinguishing hallmark of viral pneumonia in general and SARS-CoV-2 in particular. This disparity in lung injury rates could be attributed to vaccine efficacy as well as the effect of variations on the severity of COVID-19. The Delta variant, on the other hand, is more likely to develop fusion and opacity than other variants. Consolidation is an indication of extensive inflammation and corresponds to the course of the COVID-19 infection. Furthermore, the relationship between severe lung injury and oxygen therapy revealed differences between patients who required and did not require oxygen therapy over the course of treatment, which was explained by consolidation reducing the lungs’ ability to exchange oxygen; therefore, the patients require increased oxygen support. Similarly, the rate of consolidation was higher in severe and critical patients, and the rate of death from lung injury was higher in individuals who survived. Indeed, pulmonary problems after SARS-CoV-2 infection are connected with older patients and people with severe COVID-19 [29].

The proportion of patients with COVID-19 exhibiting evidence of lung injury on CT imaging is rather significant, similar to Luger's data [30]. The data also demonstrated that patients with COVID-19 with moderate to severe levels had a much higher rate of lung injury than patients with mild COVID-19 (55% compared with 3.8%, respectively). On the other hand, the patients with severe COVID-19 had a greater pulmonary involvement of all anomalies (10-25%) than the patients with non-severe COVID-19 (58% compared with 30.6%, respectively).

Severe and critical patients are of special concern because the vast majority have a poor prognosis and a high mortality rate [31,32]. Effective diagnostic prediction models that identify people at a high risk of mortality would thereby improve patient care and assist physicians in deciding the quality of treatment. The use of chest CT imaging in conjunction with clinical evaluation is critical in patient management. Furthermore, lung damage anomalies might appear in patients with conventional clinical symptoms as well as in mild or asymptomatic cases. Mair *et al.* [33] described a chest CT of a patient with lung damage but found that the result of the real-time RT-PCR test was negative. For confirmation, real-time RT-PCR testing must be repeated several times. This finding demonstrates the value of CT imaging of lung lesions in the early prognosis of COVID-19.

This is a preliminary study of lung lesions found on a CT scan in 160 patients with COVID-19, of a total of 278 patients with COVID-19 with lung CT scans. This data set presents and analyzes a variety of lung injury characteristics at three clinical levels—mild, moderate, and severe—as well as investigating the association between these features and medical variables. However, our study has several limitations. First, the sample size was only 160 patients with lung injuries, which is a potential limitation that could affect the study results. Second, when performed on a larger sample size, severe lung injury in patients with COVID-19 caused by infection with Alpha, Delta, or Omicron variants or vaccination rates may vary. Third, this study only described the lung injury of patients with COVID-19 and did not follow up the patients’ clinical development or the change in lung injury over the course of treatment.

In conclusion, the proportion of lung injury in mild and moderate patients was virtually comparable to that in severe and critical cases; however, the severity of lung injury was very low. Furthermore, patients who were fully vaccinated had less severe lung injury, whereas patients who had severe and critical clinical symptoms showed a higher rate of severe lung injury. Therefore, patients with COVID-19 fully use the CT scan for management, care, and treatment as a tool for early diagnosis and prognosis.

## Declarations of competing interest

The authors have no competing interests to declare.
